# House of cellulose - a new hideout for drug tolerant *Mycobacterium tuberculosis*

**DOI:** 10.15698/mic2016.07.515

**Published:** 2016-06-23

**Authors:** Ashwani Kumar

**Affiliations:** 1 Council of Scientific and Industrial Research, Institute of Microbial Technology, Sector 39 A, Chandigarh 160036, India.

**Keywords:** Mycobacterium tuberculosis, biofilms, cellulose, drug tolerance, thiol reductive stress, extracellular polymeric substance

## Abstract

*Mycobacterium tuberculosis* (Mtb) causes tuberculosis (TB). The treatment of TB requires administration of multiple drugs for long durations because of the unusual drug tolerance of Mtb. The phenotypic drug tolerance of genetically drug-susceptible Mtb in humans can be explained by its ability to form biofilms. Recent studies from different laboratories suggest that Mtb forms biofilms that harbour drug-tolerant bacteria. These findings have created a new area of research in the field of mycobacterial physiology. Recently, my laboratory has reported that Mtb cells organise themselves into biofilms in response to intracellular thiol reductive stress (Trivedi *et al.* Nature communications. 2016). Bacteria residing in these biofilms are tolerant towards antimycobacterial drugs. Cellulose is a key component of the extracellular polymeric substances that hold mycobacterial cells together in these biofilms. Here, I discuss the implications of these findings and new hypotheses arising from this study on the biology of Mtb biofilms.

Mtb is highly susceptible to the currently used drugs *in vitro*. However, these drugs are not effective in humans, and a combination of drugs has to be administered for at least 6–9 months for effective TB treatment. The difference between the drug susceptibility of Mtb in laboratory cultures and that in humans suggests that the currently used laboratory methods for screening of potential drugs do not simulate the physiological state of Mtb *in vivo*. Numerous hypotheses have been developed to explain the drug tolerance of Mtb in humans, including the presence of a large population of drug-unresponsive Mtb in the ‘nonreplicating persistent state’ as well as biofilms that harbour drug-tolerant bacilli. Given the diversity and complexity of the granulomas in infected humans, both hypotheses are probable and may together contribute to drug tolerance.

Although the nonreplicating persistence has been studied for several years, biofilm formation by Mtb was only recently established. Biofilm formation by Mtb is intriguing because it can explain how Mtb remains undetected by the immune system for long periods and why TB requires prolonged treatment with drugs. We are now beginning to understand the microbial physiology of Mtb residing in the biofilms. Trivedi *et al.* (2016) recently demonstrated that the intracellular thiol reductive stress (TRS) triggered by dithiothreitol can induce surface-adherent biofilm formation in Mtb cultures. Furthermore, evidence presented in this study suggests that TRS-induced Mtb biofilms harbour drug-tolerant bacteria. However, the drug tolerance is not due to the low metabolic activity of the biofilm-resident bacilli in response to hypoxia, NO, and nutrient starvation as in the case of persister cells. In fact, the bacteria residing in the TRS-induced biofilms are metabolically active. TRS-induced biofilms represent an ideal model for investigating the Mtb biofilms because they are conveniently formed in a significantly shorter duration than pellicles are.

Furthermore, the TRS-induced biofilms display some additional features of typical biofilms, such as strong adherence to the substratum and a complex architecture with several pores and channels that facilitate the diffusion of nutrients. Such features could enable Mtb to establish pulmonary and extra-pulmonary foci of infection, attached to the tissues but undetected by the immune system. Notably, the TRS-induced biofilm formation requires DNA, RNA, and protein synthesis, and the inhibition of these processes prevents biofilm formation. However, the formation of TRS-induced biofilms is unaffected by cell wall biosynthesis, suggesting that although Mtb cells are metabolically active, they are not actively dividing. In this study, the underlying transcriptome changes associated with biofilm formation were explored. Transcriptional analysis suggested that during biofilm formation, components of the cellular machinery involved in cell division and proliferation are downregulated and the resources thus spared are probably diverted towards synthesis of extracellular matrix. Transcriptome data also revealed that the biofilm-resident bacteria utilise a different set of metabolic pathways to generate NADH/NAD^+^, ATP/ADP, and NADPH/NADP^+^.

Trivedi *et al.* (2016) established for the first time that the extracellular matrix of the Mtb biofilms is composed of lipids, proteins, polysaccharides, and extracellular DNA. However, the lipids and proteins are localised in the microcolonies of the biofilms, whereas a large quantity of polysaccharides was present in their extracellular matrix. Before this study, free mycolic acids were considered the key constituents of the extracellular matrix that holds mycobacterial communities together. But this study suggested that polysaccharides are the major constituents of the extracellular matrix. The analysis of these polysaccharides further revealed that the glucose polymer cellulose is a key component of Mtb biofilms. The disruption of Mtb biofilms by cellulase and protease was a crucial observation, which suggested that cellulose and some unidentified structural proteins may be responsible for maintaining the structural integrity of the Mtb biofilms. Confocal microscopy suggested that microcolonies of Mtb are attached to the substratum through cellulose and extracellular DNA scaffolds, spread over the substratum. Because cellulose is absent in humans, I propose the use of cellulose as a biomarker to verify the presence of Mtb biofilms in humans. 

**Figure 1:**
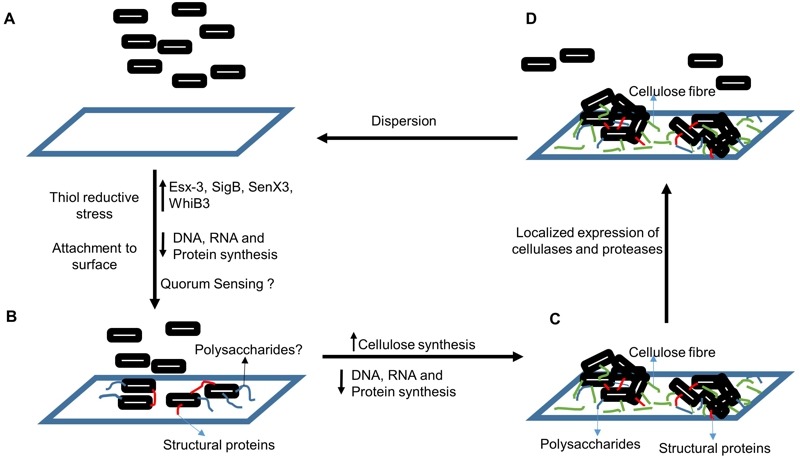
FIGURE 1: Model of Mtb biofilm formation and dispersion. **(A)** In response to TRS, Mtb cells downregulate DNA, RNA, and protein synthesis. Resources thus spared are diverted towards synthesis of polysaccharides and structural proteins that facilitate attachment with substratum and to nearby mycobacterial cells. We believe that cell density plays a critical role in this process. Therefore, quorum sensing and the underlying mechanisms could play an important role in mycobacterial decision to organise into biofilms. **(B)** If the TRS is sustained for more than six hours, Mtb cells start adhering to surface. We hypothesize that unknown structural proteins, and uncharacterized polysaccharides play a critical role in this process. **(C)** If the TRS is sustained beyond twelve hours, then cells are irreversibly attached to the substratum and start producing cellulose. By twenty nine hours, the Mtb biofilms are fully matured. **(D)** Using localized production of cellulases and proteases, degradation of the EPS at the periphery of the Mtb biofilms could facilitate escape of few Mtb cells from the biofilms. These cells could then replicate under growth conducive conditions and upon reaching critical cell density could form biofilms. **Abbreviations:** TRS - thiol reductive stress , Mtb - *Mycobacterium tuberculosis.*

Although the present study has established a new model for Mtb biofilm development and has shown that cellulose is a key component of Mtb biofilms, a number of new questions have arisen. For example, we know little about the architecture of the Mtb pellicles that represent the other established model for studying Mtb communities. The ECM of the pellicles of Mtb should be tested for the presence of cellulose or other polysaccharides. Because cellulose is now identified as a biomarker of Mtb biofilms, the detection of cellulose in granulomas and other sites of infection may enable us to determine whether Mtb cells organise themselves into communities. If Mtb communities are detected in the infected animal models or human tissue samples using cellulose as a biomarker, new strategies have to be developed for targeting Mtb biofilms by delivering antimycobacterial drugs along with cellulases to the site of infection. The next logical extension of the current study will be the identification of the cellulose synthase pathway in Mtb. Although the canonical homologues of the bacterial cellulose synthases in Mtb have not been identified through computational biology, genetic screens using chemical or transposon-mediated mutagenesis could be employed for identifying the pathway(s) involved in cellulose biosynthesis. I believe, the identification of the genetic pathway(s) that control cellulose synthesis in Mtb represents an important area of research, which will dictate our ability to inhibit the formation of the biofilms in the host. Preliminary studies in my laboratory also showed that the ECM of Mtb biofilms contains polysaccharides other than cellulose. Identifying these polysaccharides will further improve our current understanding of Mtb biofilms. I also believe that with the establishment of this new model of Mtb biofilm development, we have entered a new era of understanding the microbial physiology of this dangerous pathogen.

